# Diverse roles for CDK‐associated activity during spermatogenesis

**DOI:** 10.1002/1873-3468.13627

**Published:** 2019-10-20

**Authors:** Nathan Palmer, S. Zakiah A. Talib, Philipp Kaldis

**Affiliations:** ^1^ Institute of Molecular and Cell Biology (IMCB) A*STAR (Agency for Science, Technology and Research) Singapore Singapore; ^2^ Department of Biochemistry National University of Singapore (NUS) Singapore Singapore; ^3^ Department of Clinical Sciences Clinical Research Centre (CRC) Lund University Malmö Sweden

**Keywords:** cyclin, cyclin‐dependent kinase, meiosis, meiotic crossover, recombination, synapsis

## Abstract

The primary function of cyclin‐dependent kinases (CDKs) in complex with their activating cyclin partners is to promote mitotic division in somatic cells. This canonical cell cycle‐associated activity is also crucial for fertility as it allows the proliferation and differentiation of stem cells within the reproductive organs to generate meiotically competent cells. Intriguingly, several CDKs exhibit meiosis‐specific functions and are essential for the completion of the two reductional meiotic divisions required to generate haploid gametes. These meiosis‐specific functions are mediated by both known CDK/cyclin complexes and meiosis‐specific CDK‐regulators and are important for a variety of processes during meiotic prophase. The majority of meiotic defects observed upon deletion of these proteins occur during the extended prophase I of the first meiotic division. Importantly a lack of redundancy is seen within the meiotic arrest phenotypes described for many of these proteins, suggesting intricate layers of cell cycle control are required for normal meiotic progression. Using the process of male germ cell development (spermatogenesis) as a reference, this review seeks to highlight the diverse roles of selected CDKs their activators, and their regulators during gametogenesis.

## Graphical abstract

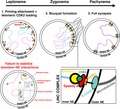

## Abbreviations

**CDKs**, cyclin‐dependent kinases

**CKIs**, CDK‐inhibitor proteins

**DSBs**, double‐strand breaks

**FSH**, follicle‐stimulating hormone

**GST**, gonocyte‐spermatogonia transition

**LINC**, linker of nucleoskeleton and cytoskeleton

**LRN**, late recombination nodule‐associated

**RPM**, rapid prophase movements

**SSCs**, spermatogonial stem cells

## Cyclin‐dependent kinases: a diverse family with numerous activators and regulators

Cyclin‐dependent kinases (CDKs) and their activating partner proteins, the cyclins, are often compared to the molecular engine and gearbox, which is utilized to drive a cell through the different phases of the cell cycle 1. This simplistic analogy is representative of the cell cycle in eukaryotic organisms of lower complexity, such as yeast. These unicellular organisms possess a single ‘engine’ (CDK) and multiple cyclin ‘gears’. The sequential association of different cyclins with the CDK promotes the activation of distinct CDK/cyclin complexes to advance through each stage of the cell cycle. In more complex multicellular eukaryotes such as mammals, the CDK and cyclin gene families have become increasingly larger, as a result of multiple gene duplication events occurring throughout eukaryotic evolution. This has allowed some CDKs and cyclins to acquire specialized functions either in addition to, or independently of their roles in cell cycle progression 2, 3. There are currently 21 CDKs, and upwards of 30 cyclin proteins described in humans and mice. Only CDK1, 2, 3, 4, and 6 in complex with an A‐, B‐, C‐, D‐, or E‐type cyclins, form canonical CDK/cyclin complexes with direct roles in driving mitotic cell cycle progression. The remaining CDKs and cyclins exhibit diverse functions and have been shown to facilitate many important cell cycle‐independent roles including the modulation of transcription and RNA splicing 4. Although much study has been devoted to the understanding of mammalian cyclins, it is clear that there are still many intricacies yet to be properly described. Recently, several ‘atypical’ cyclin family members have been identified. These proteins share limited homology with the cell cycle‐associated cyclins. Many of these have yet‐unknown functions, and their ability to bind and activate CDKs is mostly unexplored 4, 5, 6, 7, 8, 9, 10.

In addition to the cyclins, several mammalian CDKs can also be bound and activated by noncyclin CDK interactors from the Speedy/RINGO family of proteins. Noncanonical CDK/Speedy complexes are not subject to typical cell cycle regulation. Whereas the majority of CDK/cyclin complexes are reliant upon a critical activating phosphorylation to achieve full catalytic activity 11, 12, 13, CDK/Speedy complexes display activity in the absence of this modification 14, 15, 16, 17, 18. Furthermore, CDK/Speedy complexes are also insensitive to inhibition by CDK‐inhibitor proteins (CKIs), which physically bind and inactivate CDK/cyclin complexes 4, 19, 20. These unique properties are thought to allow the formation of active CDK/Speedy complexes under circumstances, whereby CDK/cyclin complexes would usually be inactivated.

In regard to the role of CDK‐associated activity in maintaining normal reproductive health, several mammalian CDKs, cyclins, and at least one Speedy protein (Speedy A) have been shown to be essential for the development and maturation of the germ cells within the male and female reproductive organs (gametogenesis). This is similarly true of members of the pRB‐E2F signaling pathway, which represents a major downstream target of CDK activity and also specific CKI proteins. Many of these proteins show preferential or heightened expression in these tissues or, in some cases, exhibit differential expression of splice isoforms 21, 22, 23, 24. In accordance with these observations, knockout mouse models of many of these proteins result in infertility due to arrested gametogenesis. Interestingly, the stage at which germ cell development is affected in these models is highly varied depending upon the protein which is knocked out. This highlights a complex network of often nonredundant interactions between CDKs, their activators, and their regulatory proteins during gametogenesis and offers a unique insight into the requirement of CDK‐associated activity for normal fertility.

## Spermatogenesis as a model system to study the importance of CDK activity during germ cell development

**Figure 1:**
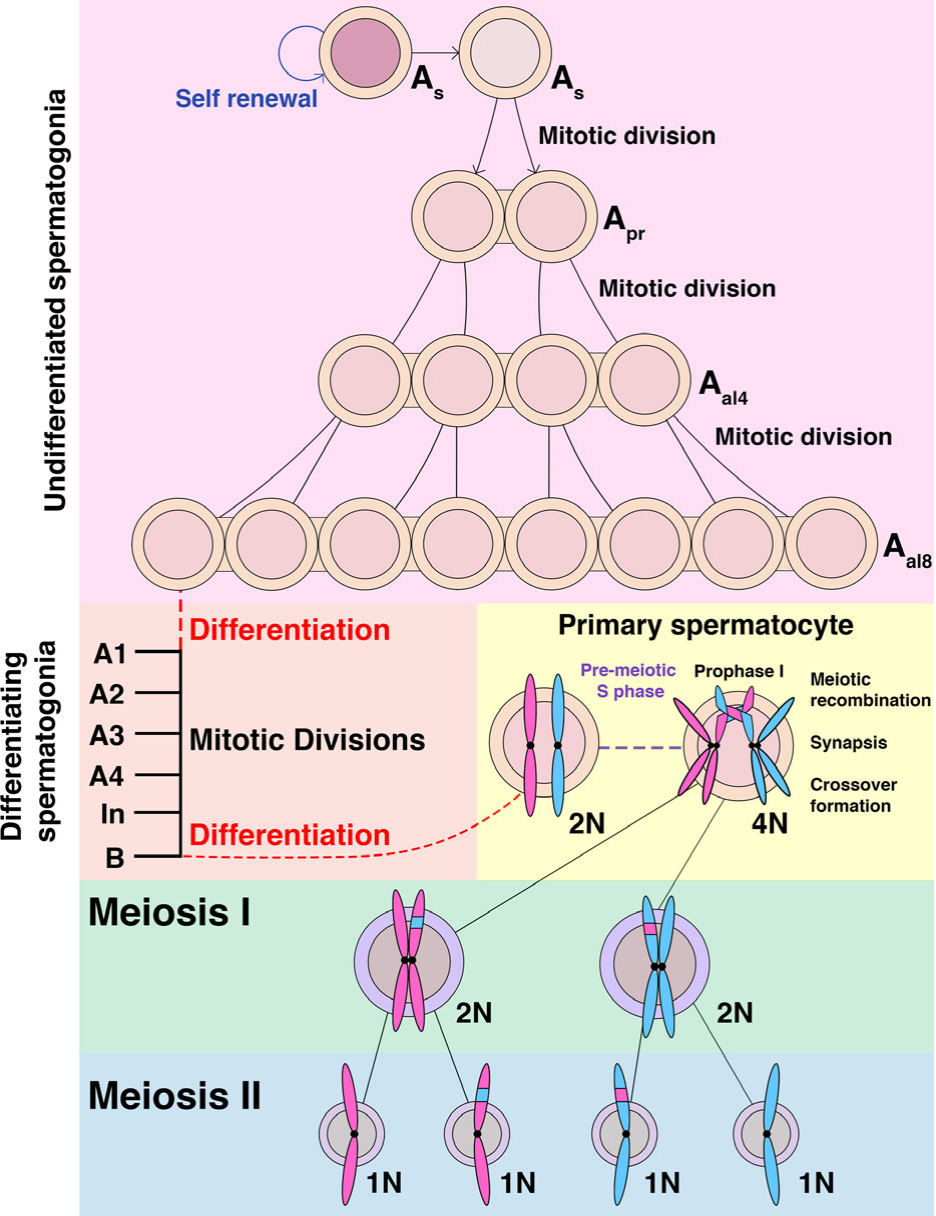
Schematic of adult spermatogenesis. Spermatogenesis is initiated by A single (A_s_) type spermatogonial stem cells (SSCs). A_s_ type SSCs are thought to be able to produce both undifferentiated A_s_ type SSCs (self‐renewal) or separate populations of A_s_ SSCs capable of differentiation. A_s_ spermatogonia undergo consecutive rounds of mitotic division to produce pairs (A_pr_) or aligned chains (A_al_) of SSCs. A_al_ SSCs represent clonal populations linked by shared cytoplasm and vary in the maximal chain length attained before further differentiation. Chains of 8 (A_al8_) or more are considered proficient to enter further differentiation. Each A_al_ spermatogonia differentiates without division to form ‘differentiating’ A1 type spermatogonia. These undergo consecutive rounds of mitotic division to produce A2, A3, A3, Intermediate (In), and B‐type spermatogonia. B‐type spermatogonia subsequently differentiate without division to form primary spermatocytes capable of meiotic division. In premeiotic S phase, diploid primary spermatocytes duplicate their DNA to become tetraploid. During meiotic prophase I, chromosomal homologs initiate meiotic recombination and synapsis allowing the formation of meiotic crossovers. Upon resolution of crossovers, homologs are segregated into two diploid secondary spermatocytes (meiosis I). Each secondary spermatocyte divides rapidly to segregate sister chromatids into two haploid round spermatids (meiosis II). Thus, four haploid spermatids can be produced from two reductional divisions of a primary spermatocyte. Not shown here is the process of spermiogenesis whereby round spermatids undergo sequential steps of differentiation to form elongating spermatids and spermatozoa.

Much of the research performed to elucidate the roles of CDK‐associated activity during gametogenesis has utilized the process of male germ cell development (spermatogenesis) as a developmental model. For such studies, female germ cell development (oogenesis) is disadvantaged by both the timing and frequency of the meiotic divisions observed in this system. In mice, the first meiotic division of female meiocytes (oocytes) is initiated in the embryonic ovary (~embryonic day E13.5 in the mouse). Meiosis is subsequently arrested prior to the onset of the second meiotic division and oocytes enter a long‐term arrest state known as dictyate 25, 26. As in humans, the generation of mouse oocytes occurs only once. Consequently, the analysis of early oocyte development and its first meiotic division must be performed during embryonic development. In contrast, once spermatogenesis is initiated in pubertal male mice (~postnatal day 14), the development of germ cells occurs in continuous waves throughout the lifetime of a mouse. This is facilitated by the continual production and maturation of male meiocytes (spermatocytes) from a self‐renewing source of spermatogonial stem cells [SSCs] 27. For normal spermatogenesis, a small pool of ‘fully undifferentiated’ or ‘primitive’ (A_s_) type SSCs must be maintained to supply a steady supply of cells competent to enter meiotic divisions. The division of these cells gives rise to either more A_s_ SSCs, which retain the capacity to self‐renew, or early differentiating spermatogonia. Differentiating spermatogonia divide successively via mitosis without cytokinesis to form first pairs (A_pr_), and then aligned chains (A_al_) of spermatogonia linked by cytoplasmic bridges. These chains can attain lengths of 16 or rarely 32 connected cells. It is generally considered that chains of at least 8 (A_Al8_) must be formed to enable further differentiation 28. Committal of A_al_ spermatogonia cells to gametogenesis occurs in the absence of mitotic division and is characterized by morphological changes as well as changes in transcription 29, 30. The resultant A1‐type spermatogonia undergo sequential mitotic divisions to become A2, A3, and then A4‐type spermatogonia. A4 spermatogonia undergo further divisions to become intermediate‐type (In) and then B‐type spermatogonia. At this stage, B‐type spermatogonia are competent to differentiate into primary spermatocytes that can enter meiosis. The stepwise nature of spermatogenesis as depicted in Fig. 1 is particularly suited to knockout studies. Here, the stage of germ cell development affected by the deletion of a single protein can be easily investigated to determine functionality.

## Meiotic prophase I: a hotbed for CDK‐associated functions

For the generation of haploid gametes, meiocytes must undergo two reductional meiotic divisions. The first meiotic division promotes the pairing and exchange of genetic material between maternal and paternal chromosomal pairs (homologs). This occurs during an extended prophase stage (prophase I) 31, 32 and is followed by the segregation of homologs of into two genetically heterogeneous daughter cells. This is followed by a second meiotic division, which is considered more comparable to mitotic divisions as it promotes the segregation of sister chromatids 33. The prolonged timeframe of meiotic prophase I, reflects the complexity of this developmental process and can be broken down into five further substages: Leptotene, Zygotene, Pachytene, Diplotene, and Diakinesis. In regard to the knockout mouse models discussed within this review, the majority of meiotic defects observed typically arise during one or more of these substages. Therefore, this section will introduce the events of meiotic prophase I in detail, in order that the meiotic functions of CDK activity described later in this review can be better understood.

During leptonema, the earliest stage of prophase I, paternal and maternal bivalent chromosomes locate and pair (synapse) with their homologous counterpart. An integral part of this process is the self‐imposed induction of double‐strand breaks (DSBs). This is mediated primarily by the DNA topoisomerase, SPO11 34, 35. SPO11‐mediated DSB formation occurs preferentially within so‐called ‘meiotic hotspots’ or ‘recombination hot regions’ of the genome. The biased nature of this process is enforced by epigenetic modifications placed proximal to the incipient break site 36, 37, 38, 39, 40, 41. These alterations are thought to create a preferential environment for the recombination machinery and are typically directed toward euchromatic regions depleted of nucleosomes 41, 42, 43, 44, 45, 46. Meiotic DSB formation results in the induction of approximately 150 and 200–300 DSBs during human and murine meiosis, respectively 34, 47, 48. Although this is a phenomenon, which occurs in all organisms that are capable of meiotic division, the number of break sites is highly variable between organisms. In mice, meiotic DSB formation is essential for proper chromosomal pairing 49, 50, 51 and is tightly integrated with the assembly of a tripartite scaffolding structure, known as the synaptonemal complex 52. The components comprising the axial/lateral elements of this structure (SYCP2/3) localize primarily to chromatin decorated with markers of meiotic DSBs, in a manner that remains poorly understood. The subsequent self‐assembly of axial filaments effectively anchors chromosome segments harboring meiotic break sites together within a single chromosomal core with the remaining chromatin organized in looped arrays tethered to these sites 53, 54, 55, 56.

Zygonema marks the onset of homolog pairing (synapsis). Here, the transverse (SYCP1) and central elements (SYCE1, SYCE2, SYCE2, TEX12, SIX6OS1) bind the axial element scaffold surrounding each homolog and bring them together in a zipper‐like fashion. This event brings meiotic DSBs close proximity with the near‐perfect DNA template of its chromosomal homolog within the context of the synaptonemal complex scaffold. Upon nucleolytic processing of DSB sites, single‐stranded 3′ DNA tails are generated and utilized to initiate homologous DNA damage repair in a process known as strand invasion 57. This effectively creates a large number of initial recombination sites (also known as early recombination nodules) 58.

An essential precondition for normal synapsis during zygonema is the tethering of telomeres to the inner nuclear envelope. Nuclear envelope tethering is stabilized by the interaction of proteins at the inner (SUN1, SUN2) and outer nuclear envelope (KASH5), in addition to additional ancillary interactors (TERB1, TERB2, MAJIN). Together, these proteins comprise the ‘LINC’ (Linker of Nucleoskeleton and Cytoskeleton) complex (59, 60, 61, 62, 63, 64, 65; for review, see 66). The LINC complex facilitates the creation of nucleocytoplasmic bridges connecting telomeres to microtubules within the cytoskeleton of the meiocyte allowing cytoskeletal forces to be transmitted via the nuclear envelope to drive rapid telomeric movements 67, 68, 69. These telomeric movements are crucial for establishing initial interactions between homologs and are thus essential for their eventual pairing. Much of the meiosis‐specific functions of the proteins discussed in this review seems to revolve around the formation and stabilization of the LINC complex and will be discussed at length later in this review.

In pachynema, chromosomal homologs achieve a state of full synapsis. At this time, numerous DNA repair complexes are recruited to chromosomal axes to stabilize and repair DNA‐intermediate structures created at recombination sites 70, 71, 72. In the majority of cases, these sites are repaired without the exchange of genetic material from its homologs (non‐crossovers). The remaining sites become stabilized in a manner which protects from this repair pathway. Instead, further processing results in the formation of crossover intermediate structures in so‐called late recombination nodules. These late recombination nodules represent the latest stages of homologous recombination repair, which occur prior to the formation of meiotic crossover sites 73. In mice, the formation meiotic crossovers resulting from the maturation of late recombination nodules occurs in a strictly regulated manner with 1–2 formed per homolog pair.

In diplonema, the synaptonemal complex lateral and central elements are disassembled, a process which is completed by diakinesis. Throughout these stages, sister chromatid arms remain closely connected via cohesins 74, but paired homologous chromosomes remain linked solely at meiotic crossover sites. These crossover sites act as anchor points, which allow bidirectional tension to be applied upon each homolog pair. This facilitates orderly alignment of homologs upon the meiotic spindle 75, 76, 77, 78, 79. Subsequent recognition and cleavage of crossover sites by structure‐specific endonucleases in anaphase I allows the release of tension on the spindle. This process is also known as crossover resolution 80, 81, 82. Successful crossover resolution allows the segregation of homologs into two daughter cells and completes the reciprocal exchange of maternal and paternal DNA at the resolved crossover site. Achieving this genetic exchange of information during meiotic prophase I is a hallmark of meiotic division and is the major determinant of heterogeneity within the genome of developing germ cells 55, 83, 84, 85, 86, 87.

## Aims

Despite extensive research delineating the various roles of the CDKs, cyclins, and Speedy proteins during gametogenesis, pertinent questions remain even surrounding the best‐characterized members of these protein families, particularly CDK2, CDK4, the E‐ and D‐type cyclins, and Speedy A. This review aims to highlight the contribution of these selected CDKs in regard to their functions during gametogenesis. Specific effort is made in highlighting where gaps in the current literature can be addressed in future studies. Although also essential for gametogenesis, the A‐ and B‐type cyclins in conjunction with CDK1 and/or CDK2 are not discussed at length in this review. For a comprehensive analysis of these proteins in relation to gametogenesis, the reader is directed to the following reviews by Chotiner *et al*. and Risal *et al.* 24, 88.

## Roles for canonical, cell cycle‐associated CDK activity during germ cell development

One of the major requirements for CDK‐associated activity during normal gametogenesis arises due to the need for mitotically dividing cells in the male and female reproductive organs. Typically, these mitotically active cell types consist of either precursor stem cells, which give rise to the meiocytes, or their respective supporting cell types, which promote meiocyte maturation. In this section, we will discuss the effects on fertility observed upon deletion of cell cycle‐associated cyclins, their associated CDKs, and the CKI proteins, which regulate the activity of these complexes. For added context, this section will also explore the effects occurring upon perturbation of downstream CDK/cyclin signaling. For simplicity, the order of discussion will follow the typical order of expression of these proteins throughout the cell cycle starting with the G1 stage D‐type cyclins and concluding with the S phase‐associated CDK2.

## Proposed roles for the D‐type cyclins in regulating spermatogonial proliferation and differentiation

During mitotic cell division, D‐type cyclins act primarily as the activating partners of CDK4 and CDK6. Here, CDK4‐ and CDK6‐associated kinase activity serves an important role in driving entry into G1 from quiescence and also maintaining cellular proliferation in actively dividing cells 89, 90, 91, 92, 93. The proliferative role of CDK/cyclin proteins, in general, is mediated through the phosphorylation of the major CDK‐substrate, RB1 (Retinoblastoma) tumor suppressor, and its related proteins RBL1 (p107) and RBL2 (p130) [collectively referred to here as pRB]. In the absence of CDK‐driven phosphorylation, these pRB proteins act to inhibit cellular proliferation by sequestering E2F transcription factor family members; effectively holding them in an inactive state. When released from pRB repression, E2F transcription factors promote cell cycle progression through the downstream transcription of genes that drive cell cycle progression 94.

Based on their cell‐specific expression during spermatogenesis, cyclin D‐associated kinase activity is thought to play a primarily proliferative role in driving the mitotic expansion of differentiating spermatogonia. During the first wave of spermatogenesis in which spermatogenic cells differentiate in a synchronous manner 95, each of the three D‐type cyclin members (D1‐D3) can be detected in A1 type spermatogonia as they enter a differentiating state. Later, during adult spermatogenesis, cyclins D1 and D3 can be detected in dividing spermatogonia during many stages of spermatogenesis, suggesting specific roles for these cyclins in driving cellular proliferation 96, 97. Although deletion of *cyclin D1* 98, 99 or *cyclin D3* in isolation does not affect fertility 100, the deletion of both *cyclins D1* and *D3* in combination leads to severe developmental defects resulting in early lethality 101. Since this early lethality precludes formal analysis of the relative requirement for cyclin D1 and/or D3 in spermatogonia, it is likely that as in many other cell types, that at least one of these proteins is required to promote cellular division in spermatogonia.

Unlike cyclins D1 and D3, cyclin D2 expression is required for normal fertility in both male and female mice. During spermatogenesis, the expression of cyclin D2 remains specifically restricted to differentiating A1‐type spermatogonia during adult spermatogenesis. It has been hypothesized that might reflect a role in the differentiation process of spermatogonia. Unfortunately, this is yet to be formally confirmed due to an incomplete analysis of the infertility phenotype in *cyclin D2^−/−^* testes.

In adult ovaries, which lack proliferating stem cells, *cyclin D2* is expressed in the granulosa cells, which support the maturation of ovarian follicles. In this cell type, cyclin D2 expression is essential for cellular proliferation in response to the follicle‐stimulating hormone (FSH) 102. Interestingly, the proliferation of the corresponding testicular cell type, known as Sertoli cells, is similarly responsive to FSH signaling 103 and also seems to be influenced by *cyclin D2* expression levels. This was best illustrated in studies of *inhibin α^−/−^* mice, which are unable to properly regulate FSH production. In these mice, additional deletion of *cyclin D2* was shown to slow the growth of gonadotropin‐dependent gonadal tumors, which are comprised of the Sertoli or granulosa cell types in males and females, respectively 104. Together, these data suggest that *cyclin D2* is a FSH‐responsive gene required for cellular proliferation in both testis and ovary. Future study is warranted to determine whether the spermatogenic defects observed in *cyclin D2^−/−^* mice arise from differentiation defects in spermatogonial stem cells or alternatively, the defective proliferation of Sertoli cells.

## CDK4/CDK6

Mouse knockouts for the kinases partnering the D‐type cyclins, CDK4 105, 106, and CDK6 107 are viable. Expression of at least one of these proteins is required for the early development of hematopoietic precursors and their combined knockout results in embryonic lethality due to the development of severe anemia 107. This is also true of mice with the deletion of all *D‐type cyclins* 108. During spermatogenesis, the maximal expression of *Cdk4* and *Cdk6* is observed in immature testes at which time the testes consist primarily of spermatogonial stem cells 109, 110, 111, 112. Although *Cdk6^−/−^* mice show no overt defects in gametogenesis, *Cdk4* deletion results in female infertility from birth and early‐onset infertility in male mice. Interestingly in regard to female fertility, the phenotype upon deletion of *Cdk4*, however, seems to be distinct from that of the *cyclin D2* knockout as normal follicular maturation could be observed in these mice with no defect seen in the proliferation of granulosa cells. Instead, postovulatory progesterone secretion was markedly impaired and fertility in these mice could be rescued by progesterone treatment 113. In regard to male fertility, a low percentage (~20%) of *Cdk4^−/−^* males are initially fertile until around 2 months of age. The spermatogenic defects seen in *Cdk4^−/−^* testes increase in severity with age and fertility in these animals is invariably lost in older mice 105, 106. The importance of CDK4 for fertility remains poorly understood. One proposal was that early‐onset infertility in male *Cdk4^−/−^* mice might occur in a comorbid manner with the development of spontaneous nonobese diabetes mellitus 114, which is known to have a negative impact upon fertility 115, 116. Unfortunately, the analysis of the *Cdk4^−/−^* spermatogenic defect has not been extended further than the histological analysis of mutant testis sections. Potential spermatogonial stem cell proliferation/differentiation defects in this model are therefore yet to be investigated 114. Additional unexplored roles for CDK4 in meiotically dividing spermatocytes have also been proposed by several groups and will be discussed later in the latter sections of this review.

## Regulation of CDK4/CDK6 activity by the INK4 class of CKI proteins during spermatogenesis

During mitotic division, control of CDK4 and CDK6‐associated kinase activity is exerted in part by the INK4 family of CKI proteins encoded by the genes of *Cdkn2a, Cdkn2b, Cdkn2c*, and *Cdkn2d* 117, 118. These proteins specifically inhibit CDK4 and CDK6 by inducing structural changes that prevent catalytic activation 119. Each of the INK4 proteins show distinct expression patterns during spermatogenesis and can be observed in either spermatogonia (p16^INK4A^, p19^ARF^), spermatocytes (p19^INK4D^), or both (p15^INK4B^, p18^INK4C^) 112, 120, 121, 122, 123. The *Cdkn2a* gene possesses distinct promoters upstream of alternate exons. This allows the expression of two distinct tumor suppressor proteins p16^INK4A^ and p19^ARF^ using different reading frames 124. In its capacity as a CDK4/CDK6 inhibitor, p16^INK4A^ prevents the phosphorylation of pRB proteins by directly binding CDK4 or CDK6, effectively repressing downstream E2F signaling. p19^ARF^ will not be discussed further here as it cannot bind to CDK4/CDK6 and is therefore not considered a CDK‐inhibitor 124. Deletion of *Cdkn2a* leading to the loss of both p16^INK4A^ and p19^ARF^ yields viable and fertile animals 125, as does specific deletion of p16^INK4A^ 126. Similarly, singular, or combined deletion of either *Cdkn2b* (p15^INK4B^) or *Cdkn2c* (p18^INK4C^) does not result in overt defects in spermatogenesis suggesting nonessential roles for these proteins 127, 128. In contrast to the other INK4 family members, deletion of *Cdkn2d* (p19^INK4D^) results in the apoptosis of primary spermatocytes 129. The severity of this phenotype is worsened by the additional deletion of *Cdkn2d* (*Cdkn2c^−/−^*, *Cdkn2d^−/−^*), suggesting that these proteins might have overlapping functions. Further analysis suggested that *Cdkn2c^−/−^*, *Cdkn2d^−/−^* spermatogonia show defects entering meiotic divisions. Resultant spermatocytes also fail to correctly undergo normal meiotic division 111. The phenotypes seen in *Cdkn2c^−/−^ Cdkn2d^−/−^* mice suggest that at least some inhibition of cyclin D‐dependent kinase activity is essential for the normal progression of spermatogenesis. Somewhat contradictory to these findings are observations that in knockin mice, whereby CDK4 (*Cdk4^R24C^*) or CDK6 (*Cdk6^R31C^*) are refractory to inhibition by the INK4 inhibitors 130, 131 are fertile, with no spermatogenic defects reported in either case 105, 132. Although compound *Cdk4^R24C^ Cdk6^R31C^* mutant mice, which are fully insensitive to INK4 inhibitors have also been generated, it was not reported whether these mice exhibit any fertility defect. As such, it remains unknown to what extent the repression of CDK4/6/cyclin D activity might be required for normal spermatogenesis.

## Regulation of the pRB/E2F pathway during spermatogenesis

During spermatogenesis, the prototypical CDK‐substrate RB1 has been demonstrated to be expressed in both spermatogonia and spermatocytes. The inhibitory CDK‐mediated phosphorylation at S795 has also been observed in subsets of spermatogonia. This indicates that active cellular proliferation is mediated by RB1 in these cells 121, 133. In contrast to a primarily anti‐proliferative role for RB1 during mitosis, RB1 in male germ cells seems to prevent differentiation. The conditional deletion of *Rb1* expression in male germ cells promotes the differentiation of SSCs, preventing their continued self‐renewal. Despite initial fertility in these conditional *Rb1* ^−/−^ animals, infertility occurs by 2 months of age due to a failure to replenish cells competent to enter meiosis 133. Interestingly, no overt meiotic defects during the first waves of spermatogenesis were observed in these animals, suggesting that RB1 is not essential for either of the two meiotic divisions. Constitutive deletion of *Rb1* results in early embryonic lethality 134, 135, 136. In contrast, the RB‐related proteins p107 and p130 are not required for viability or fertility 137, 138, suggesting that these two proteins are not sufficient to compensate for the loss of *Rb1* to ensure normal spermatogenesis. As RB1 is a repressor of E2F signaling, it is presumed that deletion of *Rb1* results in unrestrained E2F transcriptional activity. Somewhat at odds with this interpretation is that overexpression of E2F1 in testes induces p53‐independent apoptosis in the testes of transgenic mice and results in the apoptosis of both spermatogonia and early primary spermatocytes 139, 140. Such apoptotic cell death was not observed in *Rb1* conditional knockout germ cells.

Interestingly in the converse situation, mice with constitutive deletion of *E2f1* exhibit testicular atrophy with aging. This results in the loss of germ cells and subfertility by 3 months of age. This phenotype was also described to progressively worsen with age suggesting that, E2F signaling, at least via E2F1 is required for normal spermatogenesis 141, 142, 143. Similarly to the conditional *Rb1* knockout, *E2f1^−/−^* testis exhibit a depletion of self‐renewing SSCs 143. Additionally, during the first waves of spermatogenesis, the apoptotic removal of spermatogonia required for the correct establishment of germ/cell/Sertoli cell ratios 144, 145, 146 was noted to be perturbed and increased apoptosis was observed when these cells entered meiosis. E2F1 and several of the other E2F transcription factors have been described to exhibit specific localization during mouse spermatogenesis 143, 147, 148, 149, 150, both in spermatogonia and meiotically dividing spermatocytes. Despite this, the deletion of *E2f2* 151, *E2f3* 152, *E2f4* 153, or *E2f5* 154 does not cause overt spermatogenic defects when deleted in isolation. This, however, does not necessarily rule out the possibility of functional redundancy to allow compensation upon the deletion of certain E2F family members. The remaining E2F family members may modulate transcription independently of the pRB proteins as they lack a pocket protein binding domain and are, thus, less likely subject to CDK regulation 155, 156.

## Roles for CDK2 in the maintenance of SSC homeostasis

In addition to appropriate pRB‐E2F signaling, the regulation of CDK2 activity is also important for normal SSC homeostasis. During spermatogenesis, CDK2 is primarily expressed at high levels in prophase I stage spermatocytes where it has essential functions in mediating homolog synapsis (for details, see below). Interestingly, CDK2 expression has also been noted in SSCs albeit at much lower levels 157, 158. The biological relevance of CDK2 activity in SSCs has only recently been addressed via the analysis of knockin mice with mutations which either partially (*Cdk2^Y15S/Y15S^*) or fully (*Cdk2^T14A,Y15F/T14A,Y15F^*) ablate the inhibitory phosphorylatable ‘TY’ motif within the glycine‐rich ‘inhibitory loop’ 159, 160. When phosphorylated, the conformation of the inhibitory loop renders the CDK unable to phosphorylate potential substrates due to multiple stable interactions occurring between the CDK catalytic site and bound ATP 161. As such, CDK2 activity in these models is expected to become uncoupled from its regulatory phosphorylation potentially increasing its activity under circumstances when it should usually be inactivated. Both *Cdk2^Y15S/Y15S^* and *Cdk2^T14A,Y15F/ T14A,Y15F^* were reported as autosomal semidominant male infertility alleles, which cause male infertility when homozygous and less severe spermatogenic defects when heterozygous 159, 160. Although the spermatogenic defects observed in *Cdk2^T14A,Y15F/T14A,Y15F^* mice have not been investigated in detail, male infertility in the case of *Cdk2^Y15S/Y15S^* arises due to a failure of SSC differentiation. Unlike spermatogenesis in adult animals, the first round of spermatogenesis is initiated by gonocytes 162. Subsequent waves of spermatogenesis are initiated by SSCs following their differentiation from gonocytes during process known as gonocyte‐spermatogonia transition (GST) 162, 163. Intriguingly, *Cdk2^Y15S/Y15S^* SSCs inappropriately exhibit properties of gonocytes including the cytoplasmic localization of FOXO1 164, 165, suggesting that GST is impaired in circumstances whereby CDK2 cannot be inhibited via its inhibitory loop phosphorylation. Although the initial wave of spermatogenesis is initiated as normal in *Cdk2^Y15S/Y15S^* mice and is able to generate spermatocytes proficient to enter meiosis I, subsequent rounds of spermatogenesis are not observed. Adult *Cdk2^Y15S/Y15S^* testes are severely atrophic and despite the presence of mitotically proficient SSC‐like cells positive for the self‐renewal marker GFRA1 166, 167, these cells lack the ability to mature into differentiating spermatogonia. Furthermore A_al_ chains of spermatogonia in these mice were observed to inappropriately retain GFRA1 expression, which is downregulated in wild‐type spermatogonia of the same stage. These results suggest that regulation of CDK2 activity is required to appropriately complete the developmental switch between gonocytes and spermatogonia, and the resultant differentiation status of abnormal SSCs is not sufficient to support their further differentiation 168. Here, it is hypothesized that premature CDK2 activity in *Cdk2^Y15S/Y15S^* gonocytes results in the unscheduled phosphorylation of a factor required for GST. FOXO1 has previously been identified as a CDK2 substrate 169, 170, making it an interesting candidate in mediating such a transitory role in the determination of germ cell fate 168. It may be of interest for future studies to investigate whether the repression of CDK‐associated kinase activity is a requirement for normal GST and whether this is only applicable to CDK2. Canonically, the ‘TY’ motif is phosphorylated and de‐phosphorylated by the WEE1 kinase and CDC25 family of phosphatases, respectively 1, 171, 172, 173. As such, pharmacological inhibition or conditional knockout of these regulatory proteins might be useful in addressing such questions.

## Regulation of CDK activity by the CIP/KIP class of CKI proteins during spermatogenesis

In addition to the INK4 protein family of CKI proteins, knockout mice have also been generated for the CIP/KIP family of CKI proteins comprised of p21^CIP1^ (*Cdkn1a*), p27^KIP1^ (*Cdkn1b*), and p57^KIP2^ (*Cdkn2c*). This family is less specific than INK4 and is, thus, able to interact with a broader range of CDK/cyclin complexes including CDK1/CDK2 in addition to CDK4/CDK6.

The deletion of *Cdkn1a*, *Cdkn1b,* or both in combination is not associated with spermatogenic defects in male mice 174, 175, 176, 177. Although female infertility is seen in *Cdkn1b* ^−/−^ female animals, this results from hyperplasia of the supporting granulosa cells of the ovary which negatively impact the maturation of ovarian follicles 174, 175, 176. p21^CIP1^ and p27^KIP1^ also seem to play a role in restricting the proliferation of Sertoli cells in the testis in early development before these cells become mitotically quiescent. As such, testicular organomegaly is seen upon ablation of *Cdkn1a*, *Cdkn1b*, or both in combination 178, due to greater total numbers of Sertoli cells.

Interestingly, severe meiotic defects have been observed when endogenous levels of p27^KIP1^ are increased via deletion of the F‐box protein SKP2. Under normal circumstances, SKP2 is required for the ubiquitination of p27^KIP1^, and thus, in this mutant, p27^KIP1^ is degraded at a lower rate. Minimal fertility resulting from widespread apoptotic loss of spermatogenic cell types is seen in *Skp2^−/−^* males, and severe ovarian degeneration was also noted in female animals. Although a detailed analysis of the arrest stage of gametogenesis in these mice was not performed, apoptosis was noted to affect cells in most stages of spermatogenesis. Deletion of *Cdkn1b* in *Skp2^−/−^* mice restores fertility, in both male and female mice confirming that abnormally high levels of p27^KIP1^ were indeed the cause of defective gametogenesis in these mice 179, 180. As p27 ^KIP1^ has the potential to inhibit both CDK2 and CDK4‐associated complexes and both *Cdk2^−/−^* and *Cdk4^−/−^* mice display fertility defects, it is likely that these defects arise due to a repression of either one of these kinases. Unlike p21^CIP1^ or p27^KIP1^, specific expression of p57^KIP2^ has been noted during spermatogenesis in both spermatogonia and early spermatocytes 181, 182. The deletion of *Cdkn2c* leads to neonatal death in the majority of mutant mice 183, 184. The small number of *Cdkn2c^−/−^* mice that survive until adulthood exhibits immaturity of germinal tissues including testis and ovaries, which suggests that this gene might be necessary for the normal differentiation of germ cells 185. However, this phenotype was not described in detail and is complicated by the many additional phenotypic defects caused by constitutive *Cdkn2c* deletion and would likely require the generation of a meiosis‐specific conditional knockout model for any further investigation.

## Meiosis‐specific roles for CDK‐associated activity

Aside from the well‐characterized roles for the CDKs and their activating proteins during cell cycle progression, several of these proteins also exhibit meiosis‐specific functions. The standout example of this is arguably CDK2. In addition to the aforementioned role in the differentiation and fate decision process of SSCs, this kinase seems to be involved in several aspects of meiotic division, particularly during meiotic prophase I. One of the most best‐studied of these functions is promoting the normal synapsis of chromosomal homologs. In this context, CDK2 is reliant upon the expression of a non‐cyclin‐activating partner protein, Speedy A. To complicate matters, the canonical CDK2 activators, cyclin E1 and E2, are also essential for homolog synapsis. Accordingly, severe meiotic defects akin to that seen upon individual deletion of *Cdk2* or *Speedy A* can also be observed upon the complete ablation of both E‐type cyclins. The lack of functional redundancy between the Speedy A and the E‐type cyclins, despite sharing CDK2 as their most likely kinase partner, is intriguing and requires further investigation to be properly understood. The following sections will primarily describe the known synaptic defects arising from deletion of CDK2 or its activating proteins. Additional topics discussed in this section are far more speculative and include poorly defined roles for the E‐type cyclins in promoting telomere stability in addition to potential roles for CDK2 and CDK4 in regard to various stages of meiotic recombination.

## Potentiation of telomeric CDK2 binding by Speedy A during meiotic prophase I

A telomeric function for CDK2 during meiosis was first investigated due to the localization of this kinase to telomeres throughout the duration of meiotic prophase I alongside components of the multiprotein shelterin complex 157. In somatic cells, the shelterin complex forms a protective cap at telomeres, which prevents the inappropriate activation of the DNA damage response against telomeric DNA 186. During meiotic prophase I, the shelterin complex fulfill a secondary role in ‘tethering’ telomeres to the LINC complex during zygonema (see introduction for prior discussion of the LINC complex). One initial step in the formation of the LINC complex is the interaction between the core shelterin complex member TRF1 and the noncanonical CDK2 activator: Speedy A. Here, TRF1 is thought to act as a scaffold to promote the interaction of Speedy A and CDK2 at the telomeres 65. In accordance with this theory, telomeres of *Trf1^−/−^* spermatocytes show a complete failure to recruit both CDK2 and Speedy A 65. Additional evidence suggests that the loading of Speedy A to shelterin is likely to occur at the nuclear envelope, as Speedy A can be detected as multiple foci at this structure prior to the initial tethering of telomeres to the nuclear envelope. Importantly, this interaction is also a prerequisite step for the telomeric binding of CDK2, as telomeric CDK2 foci cannot be observed in *Speedy A^−/−^* spermatocytes 187. This CDK2‐recruiting role for Speedy A is supported by the observation that mutagenesis of key Speedy A‐interacting residues on CDK2 negatively impacts the ability of CDK2 to localize to telomeres 187. One key factor driving the interaction between CDK2 and Speedy A seems to be the existence of a longer meiocyte‐specific splice isoform of CDK2 (p39) 22. p39 CDK2 is a preferential partner of Speedy A in meiotic cells as opposed to the normal (≈ 33 kDa) isoform, which forms canonical complexes with cyclins 22, 187, 188. The relevance pertaining to the existence of two distinct isoforms of CDK2 during meiotic prophase is still not fully understood. However, this could potentially be utilized to compartmentalize the activities of both CDK2/Speedy A and CDK2/cyclin complexes for specific cellular processes.

## The roles of CDK2 and Speedy A in promoting stable LINC complex formation

**Figure 2:**
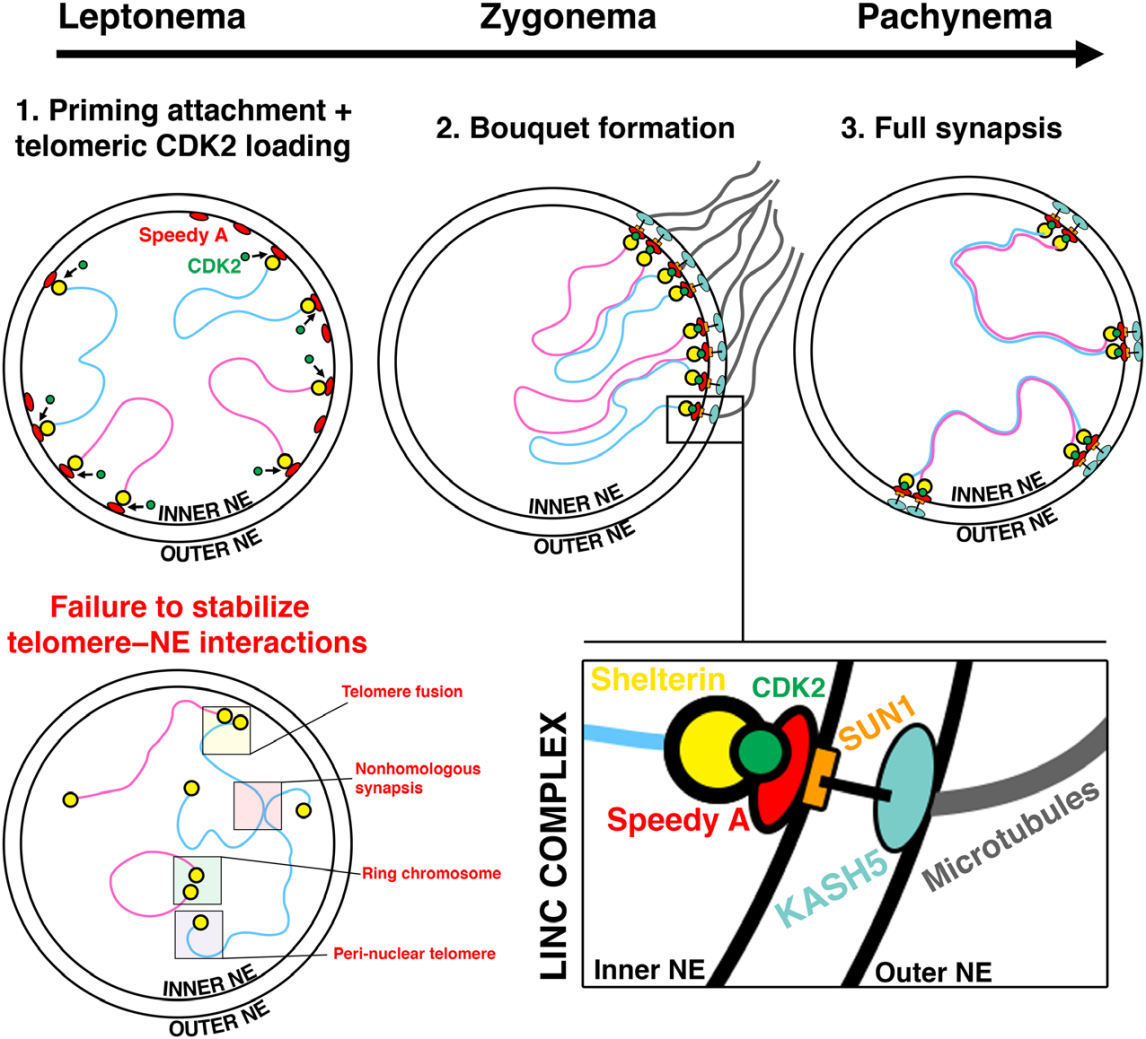
The roles of CDK2 and Speedy A in bouquet formation and synapsis. (1) During leptonema, Speedy A (red ovals) localizes to the inner nuclear envelope and interacts with the shelterin complex (yellow circles) on the telomeric ends of chromosomal homologs. This allows priming attachment or initial tethering of telomeres to the inner nuclear envelope. CDK2 (green circles) is then loaded onto telomeres in a manner dependent upon Speedy A. Here blue and pink lines are used to represent duplicated paternal and maternal chromosomal homologs, respectively; *that is,* each line contains two sister chromatids linked via their centromeres. (2) During zygonema, the interaction between shelterin, CDK2, and Speedy A is important in promoting the stable formation of the Linker of Nucleoskeleton and Cytoskeleton (LINC) complex. A simplified representation of the main components of the LINC complex is shown as a blow up below. Formation of a stable LINC complex is likely mediated by interactions between CDK2 and the inner nuclear envelope protein SUN1 (orange rectangles). SUN1 in turn is linked to the outer nuclear envelope by interactions with KASH5 (teal ovals). Cytoskeletal forces mediated by microtubules (gray) act on the LINC complex via KASH5 to drive telomere movement along the inner nuclear envelope. This allows telomeres to be polarized on the inner nuclear envelope leading to the formation of a characteristic bouquet pattern of chromosomes. (3) Bouquet formation in pachynema brings chromosomal homologs into close proximity and facilitates homolog paring (synapsis), which is completed in pachynema. An example of meiotic defect occurring upon impaired formation and/or stability of the LINC complex is shown at the bottom left hand side. These defects are characteristic of *Cdk2^−/−^* and *Speedy A^−/−^* spermatocytes and also mutants whereby shelterin or LINC complex components have been deleted.

During the leptotene–zygotene transition, complex interactions occurring between components of the LINC complex and the cytoskeleton drive the polarization of membrane‐bound proteins and the tethered telomeres. This forces telomeres into close proximity driving their clustering within a small area of the nuclear envelope, which is responsible for the characteristic ‘bouquet’ pattern of chromosomes observed at this stage 189, 190. Bouquet formation is absolutely dependent upon the prior establishment of telomere–LINC complex interactions in the leptotene stage, as this does not occur in mutants where LINC complex components or their interacting proteins have been deleted 59, 61, 62, 65, 67, 187, 191, 192. Following initial telomeric clustering, cytoskeletal forces promote rapid prophase movements (RPM), which allow chromosomes to sample their surroundings for homology in a controlled manner 193, 194, 195. This is thought to be essential for homologs to locate each other and synapse without the occurrence of inappropriate pairing between nonhomologs (nonhomologous synapsis). The involvement of CDK2 and Speedy A during bouquet formation and synapsis is depicted in Fig. 2 with examples of meiotic defects observed upon a failure to establish normal telomere–nuclear envelope interactions.

Upon deletion of CDK2 or Speedy A, stable telomere–nuclear envelope interactions are lost and telomeres are observed to become detached from the inner nuclear envelope despite initial binding. In both models, it was noted that detached telomeres remain associated with vesicles containing proteins associated with the inner nuclear membrane including SUN1, suggesting a disconnect between the telomeres and the components of the LINC complex at the inner nuclear envelope 61, 191. As a consequence, extensive nonhomologous synapsis occurs between chromosomes. In addition, telomeres engage in inappropriate inter‐ and intrachromosomal interactions resulting in telomeric fusions and the observance of ring chromosomes 61, 191. Upon failure of synapsis in *Cdk2^−/−^* and *Speedy A^−/−^* spermatocytes, chromosomes are no longer able to access their homologous counterparts to initiate the repair of meiotic DSBs by homologous recombination. Meiotically arrested spermatocytes subsequently undergo apoptosis, causing a complete block of spermatogenesis and infertility 61, 196, 197. The exact reason why CDK2 and Speedy A are required to maintain stable telomere–nuclear envelope interactions is uncertain. It is likely due to a failure to form proper interactions between the telomere and the inner nuclear envelope protein SUN1. In wild‐type spermatocytes, SUN1 localizes to telomeres tethered to the nuclear envelope during leptonema and remains bound whilst chromosomes adopt a bouquet arrangement 59, 62. In *Cdk2^−/−^* and *Speedy A^−/−^* spermatocytes, SUN1 fails to form a polarized cap along the nuclear envelope in association with its LINC complex partner KASH5 61, 62. Although interactions between CDK2, Speedy A, and SUN1 have not been confirmed *in vivo*, Ser48 within the N terminus of SUN1 has been shown to be phosphorylated by CDK2 61. Additionally, SUN1 was shown to be co‐immunoprecipitated with CDK2 *in vitro*, confirming at least that SUN1 is a worthwhile target for future studies 62. As the focal point linking the telomere and nuclear envelope, it is tempting to speculate that phosphorylation of SUN1 might stabilize its interaction with other LINC complex components, allowing telomeres to be grouped into their bouquet formation. The existence of a CDK2 substrate driving this process is supported by the fact that a knockin point mutant of *Cdk2* where catalytic activity has been ablated (*Cdk2^D145N/D145N^*) shows essentially the same phenotype as both *Cdk2^−/−^* and *Speedy A^−/−^* spermatocytes 198. This is despite normal telomeric localization of both mutant CDK2^D145N^ and Speedy A (unpublished data from the Kaldis laboratory).

This observed requirement for CDK2 catalytic activity is somewhat at odds with the observation that a truncated Speedy A protein, which is competent to bind telomeres and CDK2 but unable to activate CDK2, is able to rescue impaired telomere–nuclear envelope interactions (when electroporated into *Speedy A^−/−^* testis). This suggested that CDK2/Speedy A catalytic activity (against SUN1 or otherwise) is not required for this process 187. An alternate explanation is that catalytically inactive CDK2/Speedy A is able to maintain some degree of telomeric–nuclear envelope binding via noncatalytic interactions with SUN1 and/or its related protein SUN2. Relevant to this point is the observance that even in *Sun1^−/−^* mice, suboptimal levels of telomeric tethering and even bouquet formation can be observed although this is ultimately not sufficient for normal synapsis in pachynema 69. Further investigation into the candidacy of SUN1 as a biologically relevant CDK2/Speedy A substrate will likely require the generation of a nonphosphorylatable *Sun1* mouse mutant.

## The importance of E‐type cyclins for normal synapsis and telomere stability

Recent revelations obtained from knockout models of the LINC complex interactors TERB1, TERB2, and MAJIN have demonstrated the importance of a process termed telomere cap exchange for normal fertility. This event is thought to maintain the telomeric stability of membrane‐bound chromosomes during pachynema 64, 199. During cap exchange, telomeric DNA is transferred to a complex containing TERB1, TERB2, and MAJIN. This requires the dissociation of the shelterin complex proteins from the telomeric ends and results in the formation of a structure known as the telomere attachment plate, which integrates telomeric DNA within the inner nuclear envelope 64, 200, 201, 202, 203, 204, 205, 206, 207. The formation of telomere attachment plates can be determined via the appearance of conical thickenings at the ends of pachytene stage chromosomal axes. Interestingly, the typical formation of these structures is seemingly dependent upon the expression of the E‐type cyclins. Upon deletion of *cyclin E2*, the conical thickening of telomeric ends is compromised in a manner that can be progressively worsened upon additional deletion of one, or both copies of *cyclin E1*. In such *cyclin E* mutant spermatocytes, the failure to develop normal telomere attachment plates was associated with the observance of telomeres within the nuclear space. Furthermore, such abnormal telomeres showed reduced localization of shelterin complex components and positivity for the DNA damage marker, γ‐H2AX, suggesting inappropriate protection of telomeric DNA 208, 209. Despite such distinct telomeric defects, the E‐type cyclins do not exhibit telomeric localization during meiotic prophase, making their interaction with telomeric CDK2 uncertain 209. However, CDK2 has been shown to co‐immunoprecipitate both cyclin E1 and cyclin E2 in protein extracts from wild‐type pachytene spermatocytes. This suggests that a pool of likely nontelomeric CDK2/cyclin E is indeed present in meiotic cells 209. A functional relationship between CDK2 and the E‐type cyclins is also supported by the observation that the deletion of these proteins disrupts normal patterns of CDK2 localization during meiotic prophase. In *cyclin E2^−/−^* spermatocytes, for example, only 59% of telomeres were reported to show CDK2 binding at intensities similar to that of wild‐type controls. The additional deletion of one or both copies *cyclin E1* subsequently resulted in complete loss of CDK2 binding to the telomeres. As both the severity of telomere defects and loss of CDK2 localization were similarly affected by progressive cyclin E loss 208, 209, it is likely that the action of these cyclins creates a stable environment at telomeres for CDK2 to bind.

In addition to the loss of shelterin integrity, another possibility could be that the cap exchange is defective in the absence of E‐type cyclins. Since the process of telomeric cap exchange requires the dissociation of the shelterin complex from telomeric ends, it is possible that if this process is perturbed, uncapped telomeric ends dislodged from the nuclear envelope might be exposed within the nuclear interior. This would trigger a DNA damage response directed against the uncapped telomeres, similar to that seen upon ablation of the E‐type cyclins. Fascinatingly, cap exchange has been shown to be influenced by CDK activity as the treatment of wild‐type spermatocytes with the unspecific CDK2‐inhibitor Roscovitine 210 leads to abolished cap exchange 64. In regard to a potential CDK‐substrate mediating this process, Thr647 of TERB1 was shown to be phosphorylated during cap exchange. However, electroporation of a nonphosphorylatable TERB1^T647A^ protein into the testis of *Terb1^−/−^* mice was able to rescue otherwise defective cap exchange making the biological relevance of this phosphorylation uncertain 64.

## Potential CDK2 functions in the formation and/or designation of meiotic crossover sites

In addition to promoting homolog synapsis via its function at meiotic telomeres, CDK2 is also known to localize to late recombination nodules during meiotic prophase. In this capacity, CDK2 foci can be transiently observed to localize to 1‐2 interstitial sites associated with the chromosomal axes of each homolog pair during midpachytene stage of prophase I 157, 159, 191, 209, 211, 212, 213, 214. Here, CDK2 co‐localizes with an E3 sumo‐ligase, RNF212, and a proposed sumo‐targeting ubiquitin ligase, CCNBIP1 (HEI10) 211, 213. These proteins, together with CDK2, have been described as pro‐crossover factors. This is due to their role in the formation and maturation of late recombination nodules, which is associated with the subsequent recruitment of MutLγ (MLH1, MLH3). This is one of the final steps required for the eventual repair of recombination intermediates to form type I crossover sites 215, 216, 217, 218, 219. Late recombination nodule‐associated CDK2 foci appear independently of Speedy A, which is solely observed at telomeres during meiotic prophase I 187, suggesting that any activity of CDK2 at these sites is likely mediated by other activating proteins.

**Figure 3:**
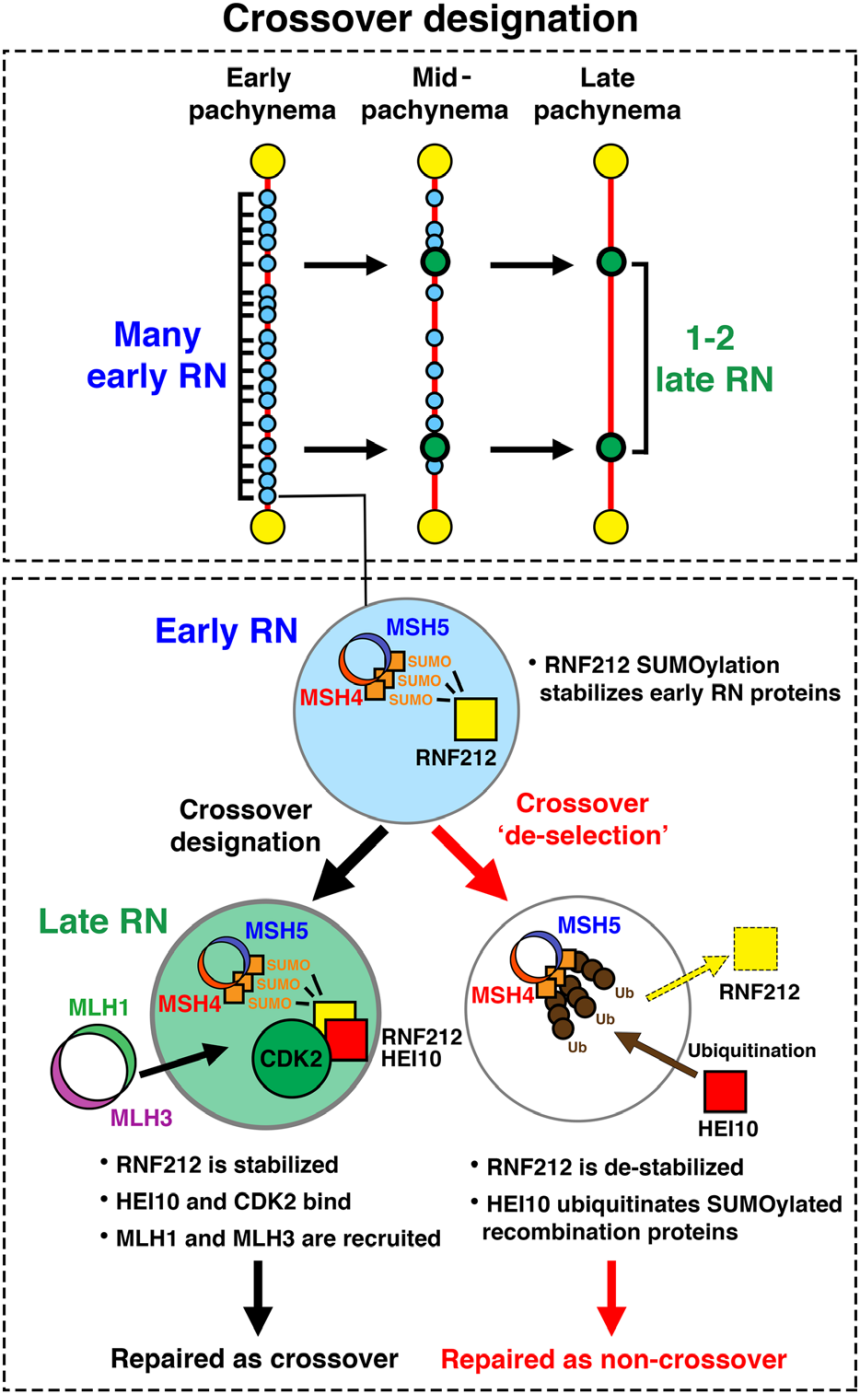
Schematic of the crossover designation process. Upper box: pattern of early and late recombination nodule formation during meiotic prophase I. During early pachynema, proteins associated with early recombination nodules (early RN) localize as many distinct foci along the chromosomal axes. Here, paired chromosomal axes are represented by a red line and early recombination nodules are represented by blue circles. By mid‐pachynema, crossover designation leads to the selection of 1 or maximally 2 early recombination nodules to mature into late recombination nodules (late RN) as shown by green circles. At the same time, nondesignated early recombination nodules are repaired and their associated proteins dissociate from the chromosomal axes. By late pachynema, only late RNs remain associated with the chromosomal axes. These mark the sites at which meiotic crossovers will form. Lower box: Events leading to crossover designation or crossover de‐selection at early recombination nodules. During early pachynema, early RNs exhibit specific localization of many proteins including the MutSγ complex comprised of MSH4 (blue ring) and MSH5 (red ring). MutSγ is thought to be stabilized by SUMOylation mediated by RNF212 (yellow square). During mid‐pachynema, one of two possibilities can occur at early RNs, crossover designation (left hand side) or crossover de‐selection (right hand side). Crossover de‐selection occurs when RNF212 becomes destabilized and dissociates from early RNs. This is thought to allow the ubiquitin ligase CCNBIP1 (HEI10) (red square) to target early RN proteins such as MutSγ for ubiquitination and degradation. These events are associated with the downstream repair of early RNs as non‐crossovers. Crossover designation occurs when RNF212 remains stabilized at early RNs. This is associated with the localization of HEI10 and CDK2. These events are characteristic of late RN formation and leads to the downstream recruitment of the MutLγ complex comprised of MLH1 (green ring) and MLH3 (purple ring). These events are required for the downstream repair of late RNs as crossovers.

**Figure 4:**
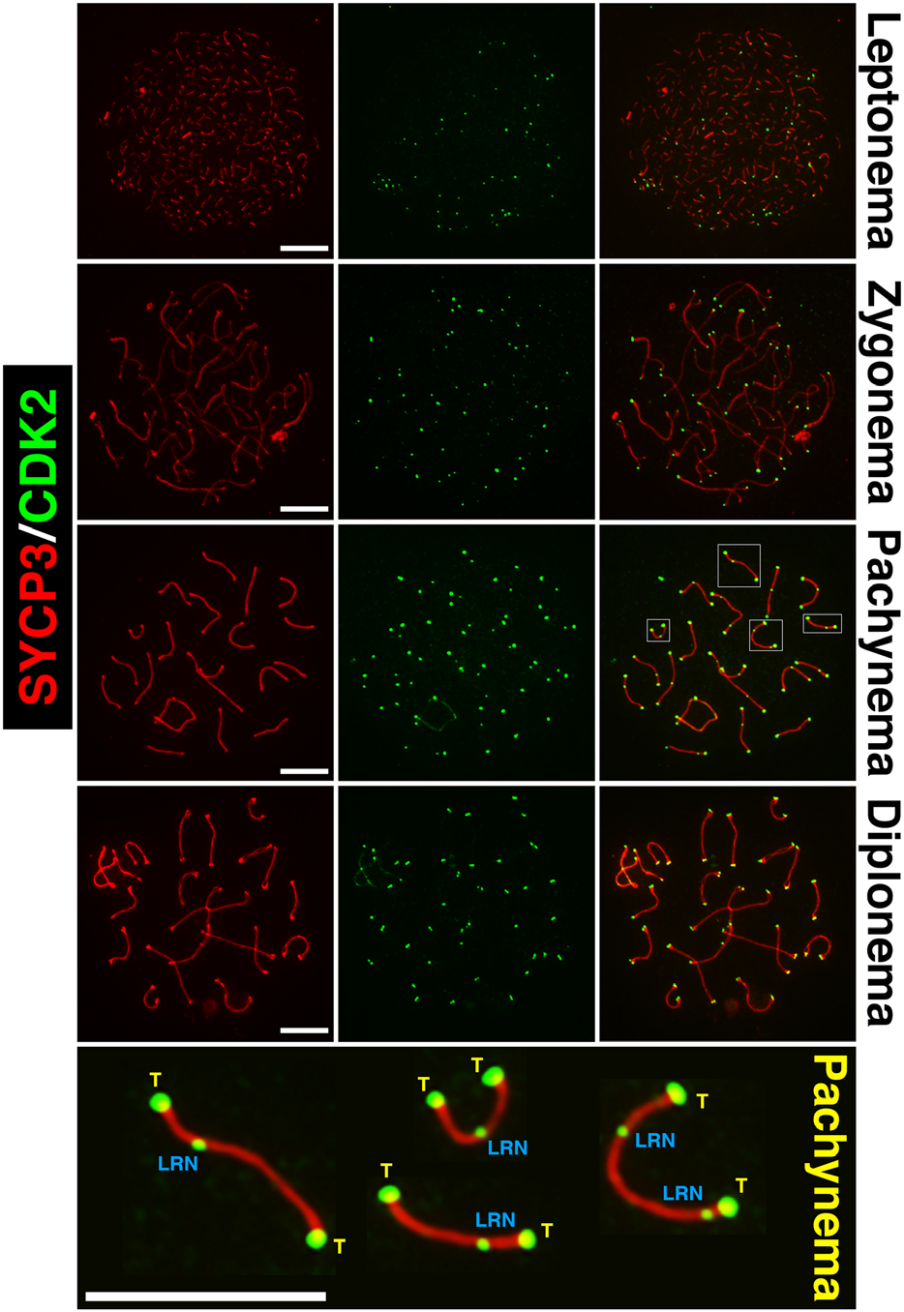
The pattern of CDK2 localization to chromosomal axes during meiotic prophase I. During leptonema, CDK2 foci (green) can be observed to localize to the telomeres of each chromosome. At this stage, chromosomal axes have not yet been formed as synaptonemal complex formation as determined by SYCP3 staining (red) is still at an early stage. During zygonema, homolog pairing is initiated and distinct axes of SYCP3 can be observed to associate with each other. At this stage, CDK2 localization is still specific to telomeres. During mid‐pachynema, intense singular CDK2 foci can be observed at paired telomeres of homologs. At this point, 1‐2 weaker but distinct interstitial foci of CDK2 can be observed marking the formation of late recombination nodules. In the lower most panel, several examples of paired homologs are shown at higher magnification to indicate both telomeric (T) and late recombination nodule‐associated (LRN) CDK2 foci. At this stage, staining can also be seen on the X‐Y chromosomes, but this is not addressed within the scope of this review. By diplonema, late recombination nodule‐associated CDK2 foci dissociate from the chromosomal axes but can still be observed as pairs of CDK2 foci at chromosomal ends. These represent the individual telomeres of each homolog, which separate upon the splitting of chromosomal axes. Scale bars for each image are shown in white and are equivalent to 5 µm in all pictures.

During early meiotic prophase, abundant RNF212 foci can be observed to localize to sites of recombination. During pachynema, a subset of RNF212 foci are able to achieve a ‘stable’ state. This is associated with the subsequent localization of HEI10. Both the increase in RNF212 foci size and the presence of HEI10 foci are considered indicators of sites selected to become late recombination nodules. They are also termed as crossover designation 211. Prior to crossover designation, RNF212‐mediated SUMOylation is thought to stabilize the MutSγ (MSH5, MSH5) complex at recombination sites, promoting their progression toward crossover‐associated repair. At smaller ‘undesignated’ RNF212 foci, RNF212‐mediated SUMOylation also seems to act as a substrate for HEI10. This allows the ubiquitination and turnover of recombination proteins, including MutSγ and RNF212 itself, at recombination sites 220. Sites depleted of MutSγ and RNF212, fail to mature, and are subsequently repaired as non‐crossovers. Therefore, the action of HEI10 can be considered as a mechanism to deselect all but ‘designated’ RNF212 foci from maturing into late recombination nodules. This effectively prevents the formation of excess meiotic crossover sites. A model of crossover designation indicating the observance of CDK2 during this process is presented in Fig. 3. This is based upon the currently known functions of RNF212 and HEI10, as reported by the Hunter laboratory 211, 213. Figure 4 depicts the locations of CDK2 over the meiotic prophase.

In addition to RNF212, HEI10, and CDK2, an atypical cyclin protein, ‘cyclin N‐terminal domain‐containing protein 1’ (CNTD1), has also been identified as a pro‐crossover factor. This protein seems to be essential for the de‐selection of undesignated crossover sites. In both *Cntd1* ^−/−^ and *Hei10* ^−/−^ spermatocytes, stable foci containing both MutSγ and RNF212 foci fail to undergo the de‐selection process in pachynema and crossover designation does not occur 213, 221. The relationship between CNTD1 and HEI10 is still not understood, but one possibility could be that CNTD1 is important for the destabilization of RNF212 at ‘nondesignated’ sites. This destabilization would then allow HEI10 to better target RNF212‐SUMOylated targets for degradation.

At present, the requirement for CDK2 localization at late recombination nodules remains unknown, but this is seemingly affected by the loss of other pro‐crossover factors. Upon singular deletion of *Rnf212*, *Hei10,* or *Cntd1*, CDK2 localization to recombination nodules is severely depleted whilst telomeric CDK2 binding remains seemingly unaffected 211, 214, 221. This indicates that these proteins are responsible or required for the localization of CDK2 to recombination nodules. As crossover designation fails to occur in each of these models, it is likely that the binding and/or action of CDK2 at these recombination nodules either occurs in parallel or directly after the observance of this process. In regard to the possible functions of CDK2 at these sites, it has previously been suggested that CDK2 might form a complex with CNTD1, which functions within the crossover designation process 221. However, at present there is no cytological evidence that CNTD1 is able to localize to recombination nodules or indeed that this protein might interact with CDK2 to form an active kinase complex. It should be noted here that the homolog of CNTD1 in *C. elegans*, COSA‐1, can localize to meiotic crossover sites 222, 223, 224. However, the comparison of COSA‐1 functions with mammalian CNTD1 is complicated by considerable differences in the process of crossover formation in *C. elegans*. For example, in this organism, MutSγ seems to function during the final stages of crossover formation and can be observed to localize at crossover sites in late meiotic prophase in a similar manner as described for mammalian MutLγ. Furthermore, although MutSγ foci are stabilized upon deletion of *Cntd1*, MutSγ becomes destabilized in *Cosa‐1* mutants 222. One model addressing potential crossover‐associated CDK2 functions suggested that HEI10 might promote the destruction of CDK2‐bound cyclin allowing it to bind late recombination nodules, possibly in complex with another crossover‐specific interactor 214. As a matter of fact, the formal gene name for *Hei10* is cyclin B1‐interacting protein 1 (*Cnnb1ip1*), which was given due to the discovery that HEI10 can interact with cyclin B1 in a yeast two‐hybrid screen 225. In addition, HEI10 is also a known substrate of cyclin B‐associated CDK activity in vitro 225 and contains several potential CDK consensus sites for phosphorylation within its C terminus. Of these sites, Ser242 (in mouse) is conserved in multiple eukaryotic species and harbors a putative cyclin binding motif upstream of this residue, making it a possible candidate substrate for CDK2 221. The importance of HEI10 for meiotic crossover formation was originally identified in an infertile, *N*‐ethyl‐*N*‐nitrosourea‐induced mutant mouse model (*Hei10^Mei4/Mei4^*) 214. This model resulted in a partial deletion of the N terminus of HEI10 within another predicted cyclin binding motif, suggesting that loss of HEI10 functions, in this case, might have been caused by the loss of cyclin interaction. To unravel the mysterious functions of CDK2 and its associated proteins at late recombination nodules, it may be a requirement to determine the network of proteins localized specifically to these sites. This might be achievable via immunoprecipitation of the crossover‐associated proteins HEI10 or RNF212 followed by mass spectrometry.

## Unexplored roles for cyclin D‐dependent kinase activity during spermatogenesis

In addition to being readily detectable in spermatogonia, CDK4 alongside its partner proteins cyclin D2 and cyclin D3 can also be detected in meiotically dividing spermatocytes 97, 112, 157. In support of a functional role for CDK4 during meiotic division, at least two independent laboratories have indicated that CDK4 is able to localize to the chromosomal axes of spermatocytes in zygonema of meiotic prophase. Here, CDK4 appears as foci in areas where homologous chromosomes have synapsed. CDK4 foci persist until mid‐pachynema at which time they can no longer be observed 157, 221. Such localization has not been reported for CDK6, suggesting that these roles during meiotic prophase might be unique to CDK4. The localization pattern of CDK4 suggests that this kinase might be required for homolog synapsis sometime after meiotic recombination has been initiated. Based on this localization pattern, it is possible that CDK4 might be required to stabilize interactions between the axial and lateral/central elements of the synaptonemal complex to promote their interaction. Many such proteins including SYCP1, SYCE1, and TEX12 have already been noted to be phosphorylated during meiotic prophase I 226, 227. Another possibility is that CDK4 might help to destabilize proteins associated with asynapsed axes to promote their dissociation upon completion of synapsis. For example, the HORMA domain‐containing proteins, HORMAD1 and HORMAD2, are prevalent on asynapsed axes but are quickly removed upon synapsis 228, 229. One further possibility could be that CDK4 might interact with complexes such as MutSγ to stabilize early recombination nodules. In *Cntd1^−/−^* mice, CDK4 foci persist at high level throughout pachynema and can be observed in late pachytene spermatocytes. This was similar to the persistence of both RNF212 and the MutSγ complex, also observed in these mutant spermatocytes. This potentially suggests a link between stabilized recombination intermediates and CDK4. If such a stabilizing role is undertaken by CDK4, other possible CDK4 interactors could be the SPO16‐SHOC2 complex and/or TEX11. These proteins are required for stable MutSγ foci formation and also appear to localize to chromosomal axes during similar stages of meiotic prophase, as described for CDK4 230, 231. To date, interactions between CDK4 and meiotic interactors have not been investigated, and the role of this kinase is still uncertain. Elucidation of the possible function of axes associated CDK4 foci will likely require an in‐depth analysis of the spermatogenic defect of *Cdk4^−/−^* mice. In regard to this question, the preparation of meiotic surface spreads from *Cdk4^−/−^* might be informative as to whether CDK4 contributes toward meiotic processes such as synapsis or meiotic crossover formation.

## Potential CDK2 functions related to the regulation of transcription in male germ cells

In addition to the known and potential meiotic roles of CDK2 discussed above, the Kaldis laboratory has recently identified a novel interaction between CDK2 and chromatin 232. This is mediated by interactions between CDK2 and the nuclear respiratory factor 1 transcription factor (NRF1) in male germ cells. Remarkably, through ChIPseq and ChIP‐reChIP approaches, we detected interactions of CDK2 with chromatin‐bound NRF1 at its target gene promoters. Despite the fact that this transcription factor is canonically associated with the regulation of mitochondrial respiration, many of the genes regulated by NRF1 in male germ cells were found to function within meiotic processes, a finding also noted by a prior study 232, 233. Although a thorough analysis of this relationship in meiotic cells was precluded by the early arrest stage of *Cdk2^−/−^* spermatocytes and the lethality associated with *Nrf1* deletion 234, we were able to observe that CDK2 was a negative regulator of NRF1 transcriptional activity. In line with this theory, we were also able to demonstrate that the DNA binding domain of NRF1 is a substrate of CDK2 and that the phosphorylation by CDK2 decreased NRF1 binding activity to chromatin. In terms of the biological relevance of this interaction, we found that the expression of one of the many NRF1/CDK2 targets, *Ehmt1,* was elevated in germ cells upon conditional deletion of *Cdk2*. This was associated with the perturbation of EHMT1‐dependent placement of H3K9me2, during the zygotene–pachytene transition. Together, these findings led us to hypothesize that the expression of CDK2 might be important to modulate NRF1 transcriptional activity during meiotic prophase, which affects the transcription of many meiotic genes including *Ehmt1*, *Msh4*, *Asz1*, *Syce1*, and *Tex19.1*. This hypothesis might be best tested in future studies via the conditional deletion of *Nrf1* in spermatocytes, utilizing a previously described *Nrf1^flox/flox^* model 233. This work further underlines that CDK2 has multiple functions in germ cell development in addition to the established function of tethering telomeres to the nuclear envelope. It will be interesting to determine the cyclin partners and the specific CDK2 substrates that regulate each of the diverse functions of CDK2.

## Outstanding questions and outlook

Knockout mouse models have long been the method of choice when ascribing functions to the various cell cycle‐associated genes. An almost exhaustive list of CDK and cyclin knockout mouse models have now been described, offering a great deal of information about the activity of CDKs and their regulatory proteins during gametogenesis. Although many of these roles have been described in the sections above, this is by no means an exhaustive list. Notable exclusions excluded from this review are CDK1, as well as the A‐type and B‐type cyclins. Currently, it is not possible to investigate the effects of *cyclin A2* or *cyclin B1* deletion due to their requirement for normal development 235, 236. Although conditional deletion of *Cdk1* in spermatocytes or constitutive deletion of *cyclin A1* has been shown to cause male infertility, these proteins primarily function to drive the exit from meiotic prophase I. Accordingly, these proteins are not required for meiotic recombination, synapsis of homologs, or meiotic crossover formation 237, 238, 239, 240. Similarly, cyclins B2 and B3 are not required during meiotic prophase. Although cyclin B3 deletion results in female infertility, this arises due to its role in triggering anaphase I exit in oocytes 236, 241, 242. There is ample space for future investigations to determine the functions of these cell cycle regulators in germ cell development and meiosis.

In regard to future research, we perceive considerable scope to uncover novel information regarding CDK functions during germ cell development. For example, many of the so‐called atypical cyclins are known to exhibit biased or heightened expression in meiotic tissues 243. Although the function of these proteins is mostly unexplored, knockout models for several of these genes have been described to exhibit fertility defects 244, 245. In a similar vein, the Speedy/RINGO family members Speedy B1a, Speedy B1b, and Speedy B3 16, 246 also exhibit high levels of expression in meiotic tissues. At present, genetic knockouts for these proteins have not been described and it is, therefore, uncertain whether these proteins might be essential for meiotic division as has been described for Speedy A.

As illustrated by this review, normal reproductive health is dependent upon the proper expression and regulation of various CDK/complexes at distinct developmental stages of germ cell development. It is our view that important questions remain surrounding at least five major topics covered in this review:

1. What is the cause of spermatogenic arrest in *Cdk4^−/−^* mice? Does this arise from defects in spermatogonial proliferation/differentiation, or from a failure to complete meiotic division?
2. Why does CDK4 localize to early recombination nodules and what might its activating partner at such sites be?
3. Why do Speedy A and the E‐type cyclins have nonredundant roles in promoting chromosomal synapsis? Is this due to their actions at different stages of meiotic prophase? Do the E‐type cyclins form a complex with CDK2 during meiotic prophase or are their actions CDK2‐independent?
4. How does CDK2/Speedy A stabilize the LINC complex to ensure proper bouquet formation and synapsis? Could this be via the phosphorylation of SUN1 or other related proteins?
5. What is the purpose of CDK2 localization to late recombination nodules? Does CDK2 partner with a canonical cyclin at these sites or alternatively, an atypical interactor such as CNTD1? Does CDK2 have a specific substrate at these sites, such as HEI10, which it must phosphorylate to promote crossover designation/maturation?

To elucidate the molecular mechanisms behind, the functions of CDKs in germ cell development will not only uncover more details about meiosis but will also help to understand the prevalent fertility issues that has been observed in the human population.

## Funding

The work in our laboratory is supported by the Biomedical Research Council, Agency for Science, Technology and Research (A*STAR) to PK, by SINGA (Singapore International Graduate Award) to NP, by the Biomedical Research Council—Joint Council Office Grant (1231AFG031 to PK); by the National Medical Research Council Singapore, NMRC (NMRC/CBRG/0091/2015) to PK, and by National Research Foundation Singapore grant NRF2016‐CRP001‐103 to PK.
